# Association study of *BCL9* gene polymorphism rs583583 with schizophrenia and negative symptoms in Japanese population

**DOI:** 10.1038/srep15705

**Published:** 2015-10-23

**Authors:** Hiroki Kimura, Satoshi Tanaka, Itaru Kushima, Takayoshi Koide, Masahiro Banno, Tsutomu Kikuchi, Yukako Nakamura, Tomoko Shiino, Akira Yoshimi, Tomoko Oya-Ito, Jingrui Xing, Chenyao Wang, Yuto Takasaki, Branko Aleksic, Takashi Okada, Masashi Ikeda, Toshiya Inada, Tetsuya Iidaka, Nakao Iwata, Norio Ozaki

**Affiliations:** 1 Department of Psychiatry, Nagoya University Graduate School of Medicine, 65 Tsurumai-cho, Showa-ku, Nagoya, Aichi-ken 466-8550, Japan; 2 Department of Psychiatry, Fujita Health University School of Medicine, 1-98 Dengakugakubo, Kutsukake, Toyoake, Aichi-ken 470-1192, Japan; 3 Department of Psychiatry, Seiwa Hospital, Institute of Neuropsychiatry, 91 Benten-cho, Shinjuku-ku, Tokyo 162-0851, Japan

## Abstract

B-cell CLL/lymphoma 9 (*BCL9*) is located within the schizophrenia (SCZ) suspected locus chr1q21.1. A recent study reported that a single nucleotide polyphormism (SNP) within *BCL9* (rs583583) is associated with negative symptoms of Schizophrenia, as measured by the Positive and Negative Syndrome Scale (PANSS), in the Caucasian population. We therefore investigated genetic association of rs583583, and its effect on negative symptoms in the Japanese patients. For association analysis, we used a Japanese sample set comprising 1089 SCZ and 950 controls (CON). Analysis of the effect of rs586586 on negative symptoms as examined by PANSS was investigated using 280 SCZ. Furthermore, for analysis of cognitive performance, we investigated 90 SCZ and 51 CON using the Continuous Performance Test (CPT-IP) and the Wisconsin Card Sorting Test (WCST) Keio version. We did not detect association between rs583583 and SCZ. Furthermore, rs583583 was not associated with PANSS negative scores or with CPT-IT or WCST cognitive tests. Considering the results of our previous study, combined with the results of the current study of rs583583, we argue that *BCL9* most likely does not harbor a common genetic variant that can increase the risk for SCZ in the Japanese population.

Schizophrenia (SCZ) is a chronic, more or less enervating illness that is characterized by impairments in cognition, affect and behavior, all of which have a pronounced bizarre aspect^1^. Delusions, and hallucinations, generally auditory in type, typically occur during the clinical course of SCZ^2^. SCZ is a relatively common disorder, with a lifetime prevalence of about 1%^3^. Although the overall sex ratio is almost equal, males tend to have an earlier onset than females, a finding accounted for by the later age of onset in those females who lack a family history of the disease^4^. Family history is the most important risk factor for SCZ, consistent with a genetic contribution to its etiology^5^ and the heritability of SCZ is estimated to be 64%^6^. Although genes relevant for SCZ or variants that may modulate risk for the disease have been identified using both linkage- and candidate-based or whole genome association studies, the genetic basis of SCZ is still unclear^7^,^8^,^9^,^10^. Furthermore, attention has recently been paid to endophenotypes in order to determine if they have a moderate size effect on mental disease such as SCZ.

The chromosome 1q21.1 (NCBI37: 145,479,806-145,564,639) region that was shown to be associated with SCZ^11^ contains several genes. In addition, about 75 percent of all children with a 1q21.1 microdeletion have delayed development, which particularly affects the development of motor skills, while the intellectual disability and learning problems associated with this genetic change are usually mild^12^. Since the Chr1q21.2 region contains several genes it is important to investigate which of these genes might be meaningful for Japanese schizophrenia.

The BCL9 protein is required for efficient T-cell factor–mediated transcription in the Wnt signaling pathway^13^. The Wnt signaling pathway influences neuroplasticity, cell survival, and adult neurogenesis^14^, and several studies have suggested that mental disorders may involve impairments in these functions^15^,^16^. Human genetic studies have reported that some BCL9 variants are associated with SCZ in the Chinese population^14^, but are not associated with SCZ in the Japanese population^17^ or with bipolar disorder in the Caucasian population^18^. A recent study^19^ reported that one SNP within *BCL9* (rs583583), may be associated with negative symptoms of schizophrenia as measured by the Positive and Negative Syndrome Scale (PANSS)^20^.

This SNP within *BCL9* (rs583583) has not been investigated in the Japanese SCZ population and was not investigated in previous genome wide association studies in Japan as previous studies used micro satellite markers or Affymetrix 100K arrays which didn’t cover rs583583^21^. We therefore firstly examined the association between rs583583 and SCZ in a Japanese case-control sample. Secondly, we explored the potential relationships between rs583583 in *BCL9* and negative symptoms determined by PANSS, as well as between other aspects of human cognitive function.

## Materials and Methods

### Participants

For SNP association analysis of rs583583 with a case-control study design, we used a Japanese sample set comprising 1089 cases (aged 47.1 ± 16.0 years, mean and standard deviation; males, 55.1%) and 950 controls (aged 44.7 ± 15.0 years; males, 51.2%). From the aforementioned sample we randomly (i.e. we did not apply any specific criteria for inclusion of the patients) selected subjects for further genetic analysis with a cross-sectional study design: (1) we analyzed the effect of rs583583 on negative symptoms as determined by PANSS using 280 cases (aged 45.3 ± 13.9 years; males 62.5%) and (2) we analyzed the effect of rs583583 on cognitive functions using 90 cases (aged 44.9 ± 14.2 years; males, 60.0%) and 51 controls (aged 24.6 ± 6.73 years; males, 64.7%), which also included some of the samples used for genotyping analysis.

Patients were included in the study if they (1) met DSM-IV criteria for SCZ, (2) were physically healthy and (3) had no mood disorders, substance abuse, neurodevelopmental disorders, epilepsy or known mental retardation on the basis of unstructured interviews with patients, their families and review of medical records. In addition, the patients’ capacity to consent was confirmed by a family member when needed. Subjects with a legal measure of reduced capacity were excluded. Control subjects were selected from the general public who had no history of mental disorders, based on questionnaire responses from the subjects themselves during the sample inclusion step, and based on an unstructured diagnostic interview done by an experienced psychiatrist during the blood collection step; this was ascertained during face-to-face interviews where subjects were asked if they had suffered episodes of depression, mania, or psychotic experiences or if they had received treatment for any psychotic disorders. Patients are selected from Nagoya University hospitals and its affiliated hospitals, while the healthy controls were mainly recruited from the hospital (medical staff) or graduate and undergraduate students of the medical school. Patients’ records were used to obtain relevant clinical information (e.g. age, Chlorpromazine (CPZ) equivalent doses). CPZ equivalent doses of antipsychotic medications were calculated based on the report by Inagaki *et al.*^22^,^23^. The study was described to all participants both verbally and in writing, and written informed consent was obtained from each participant. This study protocol was approved by the Ethics Committees of the Nagoya University Graduate School of Medicine and other participating institutes and hospitals. The study was conducted in accordance with the established ethical standards of all institutions.

### Genotyping and Data Analysis

DNA was extracted from peripheral blood according to a standard protocol^24^,^25^. For SNP association analysis of rs583583, genotyping was performed using a fluorescence-based allelic discrimination assay (Taqman, Applied Biosystems, Foster City, CA). Analysis was performed on an HT7900 instrument (Applied Biosystems) according to the standard protocol, and allelic discrimination of each sample was determined automatically by the default setting. Each 384-microtiter plate contained at least two non-template controls. We calculated the p-value for allele-wise association analyses. Significance was determined at the 0.05 level using Fisher’s exact test (two-sided). Statistical calculations were performed using SPSS v21 (SPSS Inc., Chicago, IL, USA) and Plink v1.07. The primary focus of this study was to investigate the association between rs583583 and Japanese Schizophrenic cases, especially the association with negative symptoms. Therefore, we performed post hoc power analyses using GPOWER (http://www.gpower.hhu.de/en.html) and Genetic Power calculator (http://pngu.mgh.harvard.edu/~purcell/gpc/). In post hoc power analyses, beta-1 (power) is computed as a function of alpha (p-value), the population effect size/minor allele frequency parameter, and the sample size(s) used in a study. It thus becomes possible to assess whether or not a published statistical test in fact had a fair chance of rejecting an incorrect null hypothesis. Data management was performed using the iMora-P system. This system is data management system that can be used to integrate clinical data (MRI data, neurocognitive tests, and similar) into a database, which can be used for medical research. Moreover, this system removes identifying particulars or details from medical test results for statistical and research purposes. Details regarding the iMora system are available upon request.

### Assessment of negative symptoms

To evaluate the effect of rs583583 on negative symptoms, we investigated negative symptoms using The Positive and Negative Symptom Scale (PANSS). We compared age, CPZ equivalent doses, positive symptoms scale, negative symptoms scale and a General Psychopathology Scale. We compared two groups that were divided by genotype (the homozygous major group and a group that included a minor allele) using Fisher’s exact test and a two-tailed *t*-test. The significance level in each scale of PANSS was set at p = 0.017 after Bonferroni’s correction (p = 0.05/3).

### Neurocognitive assessment

We investigated the effect of rs583583 on cognitive performance using the Continuous Performance Test–Identical Pairs (CPT-IP). and the Wisconsin Card Sorting Test (WCST). We used the CPT-IP Version Release 4.0 (NewCPT.exe, Copyright 1982–2004 by Barbara A. Cornblatt, All Rights Reserved) to assess working memory and visual sustained attention. The size of the PC monitor used for the test was 10.4 inches as each letter was at least 2.2 × 1.5 cm^26^,^27^. Stimuli were flashed on the screen at a constant rate of 1 per second, with a stimulus “on” time of 50 ms. Stimuli were four-digit numbers and were presented 150 times. In each 150-trial condition, 30 of the trials (20%) were target trials and required a response. Target trials were those in which the second of a pair of two identical stimuli appeared^26^. The outcome measure was a mean d’.

The WCST^28^ mainly assesses executive function including cognitive flexibility in response to feedback. We used a modified and computerized version of the WCST, the (Keio Version) (KWCST)^29^,^30^,^31^. The outcome measures were the numbers of categories achieved (CA), total errors (TE), and perseverative errors of Milner (PEM) and Nelson types (PEN) in the first trial. We selected outcomes in the WCST following a prior study that used KWCST as a measure of cognitive function^32^: (1) CA, which is the number of categories for which six consecutive correct responses are achieved (eight is the maximum number of categories which can be achieved), and is the sum measure of the level of conceptual shifts in the KWCST; (2) PEN which is the number of incorrect responses in the same category as the immediately preceding incorrect response (maximum of 47 perseverative errors); (3) PEM which is the number of incorrect responses in the same category as the immediately preceding correct response after the category changes; and (4) TE which is the total number of incorrect responses.

Cognitive data analysis was performed for the participants who completed both WCST and CPT-IP. We compared the homozygous major group and a group that included a minor allele within cases and within controls using Fisher’s exact test, a two-tailed *t*-test and Welch’s *t*-test. The significance level in five cognitive outcomes (CPT-IP mead d’, WCST CA, WCST PEN, WCST PEM and WCST TE) was set at p = 0.01 after Bonferroni’s correction (p = 0.05/ 5).

## Results

We did not detect any association between rs583583 and SCZ (Table 1). We investigated genetic effects of rs583583 on the PANSS, CPT-IP and WCST. There was no significant difference in clinical information (Tables 2 and 3).

## Discussion

In this study, we investigated the association between Japanese SCZ and the SNP rs583583. This SNP has been reported to be associated with PANSS negative symptoms in Caucasians but has never been investigated in Japanese SCZ. We also investigated an allele-wise effect on PANSS scores. However, using a Japanese SCZ population we could not replicate the results of the previous study with Caucasians.

Considering the statistical power (i.e. a type II error associated with a small effect size), the relatively small sample size of the current study compared to that of the previous study^19^ must be taken into account when the lack of association observed in the current study is discussed. In the current study, a post hoc calculations of statistical power using GPOWER showed that our total SCZ sample used for PANSS (n = 280) had sufficient statistical power (1-β > 80%) for an SNP with minor allele frequency (18%) (Table 1) in case of medium effect size (risk ratio > 2, based on Genetic Power calculation). For comparison odds ratio (odds ratio can estimate risk ratio for rare diseases) detected in previous study^19^ focused on rs583583 was 1.30. To further increase the statistical power, we investigated the association of rs583583 with SCZ subjects with predominant negative symptoms (PANSS negative score over the average values observed in our sample) using control subjects as a reference (Supplementary table 1). However the result was negative. Although the lack of association could be related to the small sample size and the lack of statistical power, it is of note that the Japanese population is considered to be genetically homogeneous^33^. Furthermore, isolated genetically homogeneous populations can be beneficial in genetic association studies and sequencing studies, owing to increased linkage disequilibrium (LD) and decreased allelic diversity^34^,^35^,^36^. These factors can allow a smaller sample size to be used to achieve the same statistical power as a large sample.

A possible obstacle in the identification of genetic variants for SCZ is its heterogeneous diagnostic entity, which is clinically relevant, though less appropriate for etiological and genetic research. Therefore, it was of interest to focus on alternative indicators of liability, or endophenotypes. We chose the CPT-IP that is designed to assess highly heritable traits (working memory and visual sustained attention) that are shown to be impaired in schizophrenic patients^37^. The WCST was selected in order to evaluate executive function. However we also could not detect an effect of the SNP (rs583583) on the CPT-IP and the WCST. It is of note that our phenotypic diagnosis is not based on structured interviews. Moreover, sample sizes of cognitive tests were relatively small and the results of cognitive tests may be biased.

Although we could not find any evidence that rs583583 was associated either with SCZ or with negative symptoms in Japanese patients, further studies focused on different populations are needed for comprehensive evaluation of the effect of this common SNP on SCZ risk. Moreover, other rare variants in *BCL9* should be searched for, and their effects on the pathophysiology of SCZ should be investigated. However, considering that the results of our previous study of other *BCL9* variants^17^, as well as the results of the current study of the rs583583 SNP, in which we could not detect association of BCL9 variants with Japanese SCZ, we argue that *BCL9* most likely does not harbor a common genetic variant that can increase the risk for SCZ in the Japanese population.

## Additional Information

**How to cite this article**: Kimura, H. *et al.* Association study of *BCL9* gene polymorphism rs583583 with schizophrenia and negative symptoms in Japanese population. *Sci. Rep.* **5**, 15705; doi: 10.1038/srep15705 (2015).

## Supplementary Material

Supplementary Information

## Figures and Tables

**Table 1:** Analysis of the association of rs583583 with Schizophrenia.

	**Case N**	**Control N**	**Total N**	**Case**^b^	**Control**^b^	**p-value**^c^	**Odds ratio**	**L95**^d^	**U95**^d^	**HWEp**^e^
rs583583 C>T (Chr1:147611315^a^)	1089	950	2039	0.187	0.202	0.24	0.91	0.78	1.07	0.21

^a^Based on NCBI 37.

^b^Minor allele frequency (MAF): The MAF in the Japanese population according to the 1000 GENOMES project website (http://www.1000 genomes.org) is 0.202 and the MAF in the total population is 0.298.

^c^Fisher’s exact test.

^d^Lower (L) and upper (U) 95% confidence intervals

^e^Hardy-Weinberg equilibrium test, p-value in control.

**Table 2:** Analysis of the association of PANSS negative symptoms with rs583583 alleles.

	**Schizophrenia**		
	**C/C (n = 197)**	**T carriers (n = 83)**	**p-value**^a^
male/female	122/75	53/30	0.89^b^
Age (years)	45.2	45.0	0.46
	13.6	14.9	
CPZeq (mg/day)	626.4	627.2	0.99
	431.9	474.4	
Positive symptom (0-42)	16.7	16.3	0.65
	6.4	5.2	
Negative symptom (0-42)	19.4	19.4	0.98
	6.5	6.3	
General (0-96)	37.9	36.3	0.22
	11.0	9.5	
Results are shown as means and standard deviation.			

^a^t-test (unless noted otherwise).

^b^Fisher’s exact test.

**Table 3:** Cognitive performance vs. rs583583 alleles.

	**Schizophrenia**			**Healthy controls**		
	**C/C (n = 58)**	**T carriers (n = 32)**	**p-value**^a^	**C/C (n = 27)**	**T carriers (n = 24)**	**p-value**^a^
male/female	36/22	18/14	0.59^b^	19/8	14/10	0.37^b^
Age (years)	44.6	45.7	0.73	24.8	24.5	0.88
	14.8	13.3		7.5	5.9	
CA	3.4	3.0	0.40^c^	5.7	5.8	0.94
	2.0	2.4		0.4	0.4	
PEN	7.1	8.0	0.52	0.6	0.4	0.38
	6.9	6.8		0.8	0.7	
PEM	4.6	5.6	0.56	0.4	0.1	0.05^c^
	7.9	6.5		0.6	0.3	
TE	20.8	23.3	0.28	10.7	10.8	0.81
	9.4	11.2		2.0	1.7	
CPT-IP 4 digit mean d'	1.3	1.3	0.96	2.7	2.7	1.00
	0.9	0.8		0.7	0.8	

Results are shown as means and standard deviation.

^a^t-test (unless noted otherwise).

^b^Fisher’s exact test.

^c^Welch’s t test.
