# Supplementary material for: Association study of BCL9 gene polymorphism rs583583 with schizophrenia and negative symptoms in Japanese population
Source: Sci Rep. 2015 Oct 23;5:15705. doi: 10.1038/srep15705 (PMC4616162; doi:10.1038/srep15705)
Supplement: Supplementary Information [file srep15705-s1.doc]

Supplementary data for

Association study of *BCL9* gene polymorphism rs583583 with schizophrenia and negative symptoms in Japanese population

Hiroki Kimura^1,*^, Satoshi Tanaka^1,*^, Itaru Kushima^1^, Takayoshi Koide^1^, Masahiro Banno^1^ , Tsutomu Kikuchi^1^, Yukako Nakamura^1^, Tomoko Shiino^1^, Akira Yoshimi^1^, Tomoko Oya-Ito^1^, Jingrui Xing^1^, Chenyao Wang^1^, Yuto Takasaki^1^, Branko Aleksic^1,**^, Takashi Okada^1^, Masashi Ikeda^2^, Toshiya Inada^3^, Tetsuya Iidaka^1^, Nakao Iwata^2^ and Norio Ozaki^1^

^*^ Equal contribution

^1^ Department of Psychiatry, Nagoya University Graduate School of Medicine, 65 Tsurumai-cho, Showa-ku, Nagoya, Aichi-ken 466-8550, Japan

^2^ Department of Psychiatry, Fujita Health University School of Medicine, 1-98 Dengakugakubo, Kutsukake, Toyoake, Aichi-ken 470-1192, Japan

^3^ Department of Psychiatry, Seiwa Hospital, Institute of Neuropsychiatry, 91 Benten-cho, Shinjuku-ku, Tokyo 162-0851, Japan

**Corresponding author:

Branko Aleksic, MD, PhD

Associate Professor

Department of Psychiatry, Nagoya University Graduate School of Medicine

65 Tsurumai-cho, Showa-ku, Nagoya 466-8550, Japan

Tel: +81 52 7442282; Fax: +81 52 7442293

E-mail: branko@med.nagoya-u.ac.jp

**Supplementary Table 1.** Association study of rs583583 and schizophrenia with strong negative symptom.

a. Based on NCBI 37.

b. Minor allele frequency (MAF): *MAF in Japanese population from the 1000 GENOMES project (*[*http://www.1000genomes.org*](http://www.1000genomes.org/)*) is 0.202 and MAF in total population is 0.298*

c. Fisher’s exact test

d. Lower (L) and upper (U) 95% confidence intervals

e. Hardy-Weinberg Equilibrium test p-value in control
